# Genome Dynamics and Evolution of Multiple-Drug–Resistant Bacteria: Implications for Global Infection Control Priorities

**DOI:** 10.1093/infdis/jiab456

**Published:** 2021-09-22

**Authors:** Sabiha Shaik, Arya Suresh, Niyaz Ahmed

**Affiliations:** 1 Pathogen Biology Laboratory, Department of Biotechnology and Bioinformatics, University of Hyderabad, Hyderabad, India; 2 International Centre for Diarrheal Disease Research, Bangladesh, Dhaka, Bangladesh

**Keywords:** genetic variation, fitness advantage, multidrug resistance, enteric bacteria, plasmids, AMR surveillance

## Abstract

Genomics-driven molecular epidemiology of pathogenic bacteria has largely been carried out through functionally neutral/inert sequences, mostly entailing polymorphic gene loci or repetitive tracts. However, it is very important to harness phenotypically relevant markers to assign a valid functional epidemiological context to tracking of pathogens. These should include microbial acumen to acquire multiple drug resistance (MDR), their physiological coordinates with reference to clinical or community-level dynamics of incidence/transmission, and their response or refractoriness to the activated immune system. We propose that multidimensional and multicentric approaches, based on diverse data integration coupled with comparative genomics and functional molecular infection epidemiology, would likely be successful in tracking the emergence and spread of MDR pathogens and thereby guiding the global infection control strategies in a highly informed manner.

Molecular epidemiological tracking of pathogenic bacteria has traditionally been accomplished through functionally neutral or inert sequences such as insertion elements, duplicated spacers, intergenic regions, or the variable number of tandem repeat loci that are spread throughout bacterial genomes. However, it is not appropriate to ignore the functional considerations of the traits that individualize isolates and define their lineages. Some of the lineage-specific characters of bacteria have a direct relevance to their propensity, for example, to acquire fitness advantages such as drug resistance. Whole-genome sequence-based epidemiology, therefore, is extremely important to provide a holistic approach based on several different genotypic and phenotypic coordinates of bacteria. It is especially relevant and practical when the costs of genome sequencing are steadily declining. Further, each new genome, in the case of those bacteria that have an open pangenome, could provide an opportunity for finding new genes and pathways and thereby new functional markers [1]

This brief report is aimed at the discussion of the above ideas in the light of our previous and ongoing works entailing high-throughput genomics and functional epidemiology of multiple drug resistant bacteria studied from different settings in the two South Asian neighbors, India and Bangladesh. As a part of this review, we also discuss the recent analyses from our research group that have previously highlighted an extensive trajectory of phenotypic resistance in clinical isolates over a period exceeding 14 years, as presented in the 15th Asian Conference on Diarrhoeal Disease and Nutrition, 2020, as well as of the genome dynamics of some clinically significant members of the Enterobacteriaceae family, namely the *Vibrio cholerae* [2], *Helicobacter pylori* [3], and *Escherichia coli* pathogens [4–8] and their trajectory of fitness advantages. Furthermore, we discuss the evolutionary mechanisms of antimicrobial resistance (AMR) [9] and the diarrheal disease burdens due to the above-mentioned pathogens, and others such as *Shigella* [10]. We also reflect upon the recombination-based mechanisms of bacterial evolution [11] and inference from the analysis of a knowledge base of plasmids, encompassing different compatibility groups entailing enteric and nonenteric pathogens and their evolutionary relationships based on shared gene contents, diversity, and plasticity (Figure 1). This might be relevant to develop a data science approach to AMR prediction based on global plasmid repertoires and to further understand the propensity of bacteria to acquire, confer, disseminate, and/or shuffle/shuttle fitness traits [11] across different species and lineages of pathogenic and environment-dwelling species.

**Figure 1.**
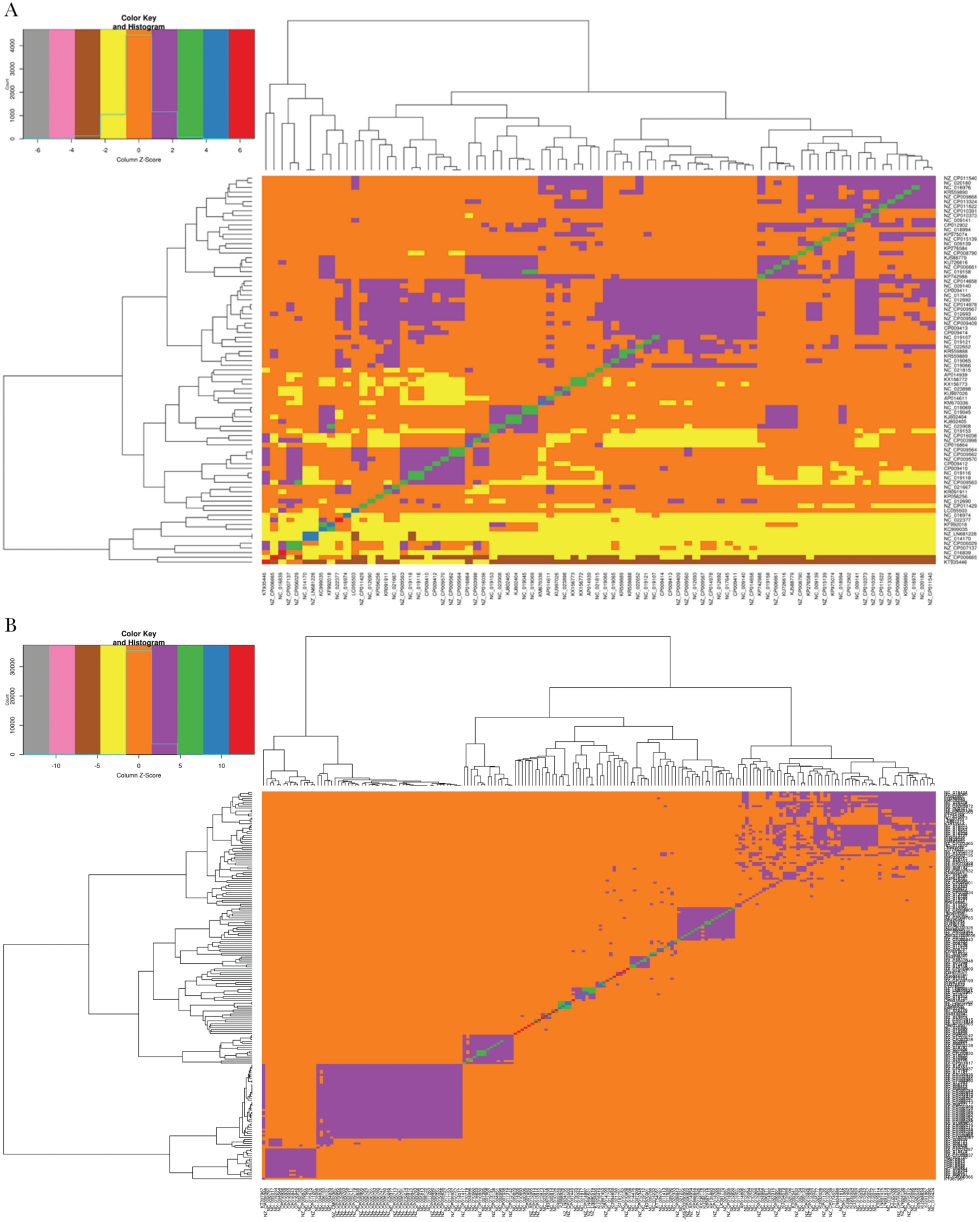
Analysis of resistance genes carried on plasmids of different incompatibility groups. The Pearson correlation among the strains based on their gene content entailing different plasmid incompatibility (Inc) groups was determined [12, 13]. Hierarchical clustering of the strains using hclust function of heatmap.2 module in R is shown for IncA/C2 (*A*) and IncF2 (*B*).

Discussion of these ideas, as presented herein, would enlighten us on the sheer prowess of bacteria to evolve and spread with new fitness advantages such as multiple drug resistance (MDR) phenotypes of different kinds. It is believed that functional molecular infection epidemiology (FMIE) approaches based on multidimensional and multicentric strategies, as mentioned above and as discussed in detail herein, would likely be successful in targeting the spread of MDR pathogens and guiding the global infection control priorities and policies.

## GENETIC VARIATION ENTAILS FITNESS ADVANTAGE

Pathogenic bacteria evolve and retrofit their genomes in response to evolutionary constraints and gain fitness advantage in different settings. Horizontal gene transfer (HGT)-mediated acquisition of DNA, genome reduction by deletions, as well as mutations and genome rearrangements form the pivotal mechanisms of evolution of different bacterial species, which occur in response to exposure to different growth conditions and selection pressures offered by the colonized niches [11]. The use of antibiotics in both clinical settings and the environment can be a major selection force for the development of resistance-conferring mutations and clonal expansion of drug-resistant bacterial populations, and can also promote the fixation of horizontally acquired resistance genes or mutations [9]. Such selection pressures could be driving the evolution and expansion of major gastric/enteric pathogens such as *V. cholerae* [2] *H. pylori* [3], *E. coli* [4], and drug-resistant *Shigella* [10].

*E. coli* evolves into different pathotypes associated with specific diseases mainly by acquisition of mobile genetic elements (MGEs) such as plasmids, phages, integrons, and pathogenicity islands through HGT [11]. In contrast, genome minimalism forms an important mechanism in the evolution of different mycobacterial species with defined host preferences and, as a result, their population structure signifies high clonality with rare or no HGT events [11]. Genomic islands that aggregate into the bacterial chromosomes play pivotal roles in the evolution of *H. pylori.* Notably, the pathogenicity island *cag*PAI displays a geographically conserved arrangement; some of the candidate genes and those encoding the outer membrane proteins and putative restriction modification systems, mostly localized in the plasticity zones of *H. pylori,* display a differential evolution pattern with a notable lineage/strain-specific prevalence [3].

HGT has been found to be responsible for the acquisition of MGEs harboring antibiotic resistance genes and leading to the phenotypic AMR in pathogenic bacteria. Thus, it is important to study the roles of MGEs in disseminating antibiotic resistance genes among the clinically significant pathogens to curb further spread of AMR [14].

The emergence of AMR in clinically relevant pathogens also warrants the development of novel antimicrobial-targeting strategies by understanding and exploiting the bacterial metabolic processes linked to survival mechanisms. For example, the gene *cgt* which encodes cholesterol-α-glucosyltransferase involved in the cell wall biosynthesis in *H. pylori,* could be an attractive target to reverse phenotypic resistance in *H. pylori* [15].

## DIFFERENT MECHANISMS EMPLOYED BY MDR PATHOGENS

Natural competence for HGT, the extent of selection pressure of antibiotics, and the rate of point mutations in the target gene could determine the potential of bacteria to acquire AMR. *V. cholerae,* a naturally competent bacterium with high genetic plasticity that allows for the genome alterations in response to selection pressures [2], is emerging with high rates of MDR [16]. A study involving *V. cholerae* from India demonstrated that 99% of the investigated isolates were MDR, and 17.2% were extensively drug resistant, and the association of the resistance genes to MGEs such as plasmids, integrative conjugative elements, and transposases could be the main culprit [16]. The pattern of AMR evolution in *V. cholerae* correlates with the extensive use of each antibiotic, the bacterium being eventually resistant to almost all the routinely used antibiotics including imipenem, meropenem, polymyxin, macrolides, aminoglycosides, doxycycline, aztreonam, and ceftazidime [17]. Antibiotic inactivation, target site replacement/protection/modification, altered membrane permeability, the expulsion of antibiotics from the bacterial cells, as well as resistance by virtue of being devoid of the antibiotic target site constitute the modes of resistance bacteria employ against antimicrobial compounds [17]. Our previous observations demonstrated high numbers of AMR genes as a part of genomic investigations of globally distributed and epidemiologically successful sequence types (STs) of *E. coli* such as ST131, ST38, ST405, and ST648 [4]. In another study, we observed that *E. coli* isolates that carry the genotoxin encoding *pks* island displayed lower phenotypic AMR as well as lower prevalence of AMR genes (especially the specific AMR determinants such as target inactivation/modification genes) [18].

## ROLE OF MOBILE GENETIC ELEMENTS IN THE SPREAD OF AMR

Plasmids as extrachromosomal genetic elements that confer key phenotypic properties to the host bacteria, such as virulence and antibiotic resistance. Through recombination, plasmids facilitate a rapid exchange of genetic information leading to the preponderance of phenotypically diverse bacterial populations [11], some with AMR. Co-occurrence of plasmid-encoded genes, which are responsible for resistance towards a broad spectrum of antibiotics, have established the pivotal role of plasmids in the dissemination of AMR. Previous studies from our research group have ascertained the role of these transmissible plasmids in the dissemination of certain AMR genes, such as the New Delhi metallo-β-lactamase (NDM) genes through conjugation experiments [7]. AMR genes belonging to multiple classes were associated with plasmids of the IncF family whereas certain plasmids of the IncI type showed association with extended-spectrum β-lactamases (ESBLs). Plasmids belonging to IncL/M and IncK types have been observed to show strong association with certain AMR genes such as *bla*_OXA-48_ and *bla*_CMY-2_/*bla*_CTX-M-14_, respectively [19].

The understanding of plasmid-based epidemiology mainly stems from the determination of phylogenetic relatedness of conserved backbone loci encoding replication and mobility functions, which are also commonly used for replicon and mobility function typing, respectively. Lately, the flood of microbial genomes [1] also led to the availability of large datasets of plasmids, providing the opportunity for high-resolution genomic epidemiology [12, 13]. However, it was observed that the plasticity of plasmid genomes is remarkably very high and even the conserved backbone loci displayed phylogenetic discordance. Therefore, alternate approaches like exploiting the gene content sharing patterns are needed to better comprehend the evolution and epidemiology of plasmids. In some of our previous studies, as presented elsewhere [12, 13] and as explained herein (Figure 1), we attempted to understand the diversity observed in the gene repertoire of a total of 548 plasmids belonging to 5 different replicon types to gain insights into their evolution. Conserved core gene clusters could not be identified for IncFIB and IncFII [12, 13]. The lack of conserved gene clusters, even at 60% sequence identity in the case of the IncF family, indicated extraordinary diversity and phylogenetic discordance of key backbone loci in these plasmids. It was also previously reported that various types of Inc plasmids are associated with the global dissemination of AMR coordinates such as *bla*_CTX-M-15_ (IncFII), ESBL, plasmid-mediated AmpC (IncFII), and *bla*_CMY-2_ (IncA/C) [19]. Such an understanding of the plasmid diversity and discordance would likely help in developing the plasmid-based epidemiology of AMR. The conserved gene clusters identified in IncA/C2, IncN1, and IncI1 mostly encoded replication and mobility functions, although the exact count of these elements differed among them (Figure 1) [12, 13]. The clustering of isolates based on the correlation of gene content sharing patterns among strains of each replicon type also indicated higher diversity in both the IncF family replicon types when compared to others. Although the overall observed diversity was very high in IncF family plasmids, a significantly close clustering, revealing a highly shared gene content among plasmids belonging to certain source organisms like *Klebsiella pneumoniae, Yersinia pestis,* etc., was identified. In contrast, among plasmids belonging to replicon types IncA/C2, IncN1, and IncI1, the overall observed diversity was low, revealing therefore a more similar gene content irrespective of the source organism of the plasmids [12, 13]. The evolution of such host specificity and genetic relatedness observed among plasmids of certain organisms belonging to the IncF family warrants more investigation. Further, with the availability of a larger set of complete sequences of plasmids in future, deeper insights can be gained in this direction.

## POSSIBLE SOURCES FOR TRANSMISSION OF AMR IN DEVELOPING COUNTRIES: INDIA AND BANGLADESH

Tracking the emergence, prevalence, and spread of MDR organisms among the populations is crucial in devising control strategies to curb AMR. Identifying the AMR gene flow from institutionalized or unorganized industry settings is imperative, especially in developing countries such as India and Bangladesh, which are densely populated [12]. Treatment of MDR bacteria is usually cumbersome and the ones from nosocomial infections are further difficult to treat given their resistance to a wide spectrum of antibiotics. The presence of NDM-producing *E. coli* among the strains collected over a period of 6 years from a hospital setting in India was previously analyzed by our research group, wherein the whole genome sequence analysis of these superbugs revealed phenotypic resistance towards a broad spectrum of antibiotics from different classes [7].

While many such studies have reported the presence of MDR bacteria from clinical samples from the countries of the global south, such as India [12, 16, 17], a notable study reported that wastewater from hospitals could be a possible source for spreading NDM-1–producing bacteria into nearby community areas of Dhaka, Bangladesh, highlighting the concern for spreading AMR through liquid waste [20]. High prevalence of MDR *E. coli* strains was also reported from the drinking water samples collected across the densely populated Rohingya camps in Bangladesh [21]. These studies warrant the need for better surveillance and interventions to check the spread of MDR strains in the municipal or refugee settings or in the community as a whole.

Food and livestock industries known for their routine use of antibiotics are perhaps the major contributors to the spread of MDR strains through the food chains. Our previous studies on whole-genome characterization, as well as comparative in vitro and in silico resistome profiling of MDR *E. coli* and *Helicobacter pullorum* strains isolated from broiler and free-range chickens from India have demonstrated resistance towards more than one class of antibiotics [22, 23]. In these studies, bacterial isolates from poultry were found to be genetically comparable with the human pathotypes, thereby strengthening the assumption of a zoonotic carriage of AMR [22, 23].

## CONTRIBUTION OF ENVIRONMENTAL FACTORS TO THE EMERGENCE OF AMR

In addition to increased selection pressures due to rampant antibiotic usage [17], a higher incidence of AMR is also linked to multiple other factors such as climate change, human migration, and population density [21]. A recent study described that an increase in local temperature by 10°C was associated with an increase in rates of antibiotic resistance of about 4.2%, 2.2%, and 2.7% for *E. coli, K. pneumoniae,* and *Staphylococcus aureus,* respectively, involving multiple antibiotic classes [24]. This indicates the impact of climate on the emergence of MDR. The impact of local temperature on HGT, bacterial growth, carriage, and transmission of resistance determinants, as well as seasonal infection patterns, could play a role in the observed patterns of association between AMR and local temperatures [24]. The study also observed a correlation between an increase in population density, as well as increased antibiotic prescriptions, and increased antibiotic resistance patterns [24].

## THERAPEUTIC IMPLICATIONS OF EMERGING AMR

Emerging AMR to conventional antibiotics also demands prospecting for other reliable strategies, including the use of phage therapy, antimicrobial peptides, and bacteriocins, as well as methods such as alteration of gut microbiota and use of predatory bacteria, which could potentially reduce reliance on antibiotics [25]. Phages that are capable of infecting bacterial hosts can be used as pharmaceutical agents of high specificity. The phage lytic cycle, which culminates at bacterial cell lysis, is suggested to be potentially useful in devising therapeutic strategies against pathogens [25]. Antimicrobial peptides (AMPs) such as colistin and polymixin B mainly interact with and disintegrate the bacterial cell wall and membrane, which form a part of the primary defense mechanisms employed by organisms against pathogens. Bacteriocins (eg, nisin) are smaller AMPs with higher selectivity and stability. Probiotics, which are the commensal bacteria capable of outgrowth and competitive exclusion of the pathogenic strains and restoration of the gut microbial balance, are safe and promising alternatives to antibiotics [25]. Furthermore, as an alternative therapeutic approach, healthy microbiota can also be introduced from a donor to a patient’s gut [25]. Antibodies that can directly act against pathogens or indirectly neutralize virulence factors are under development as alternative antibacterials. Predatory bacteria such as *Bdellovibrio* and similar organisms can enter inside Gram-negative pathogens, multiply, and eventually cause the degradation of the prey cells, and thus form potential antibacterial candidates [26]. The various alternative therapeutic interventions are at different stages of research and development; with high specificity of these therapeutics, testing and validation against a wide range of candidates might be essential [25]. Combination of such multiple therapeutic approaches, and coupling them with conventional antibiotic treatments, could also be explored towards combating the growing AMR.

## THE EPILOGUE

**Figure 2.**
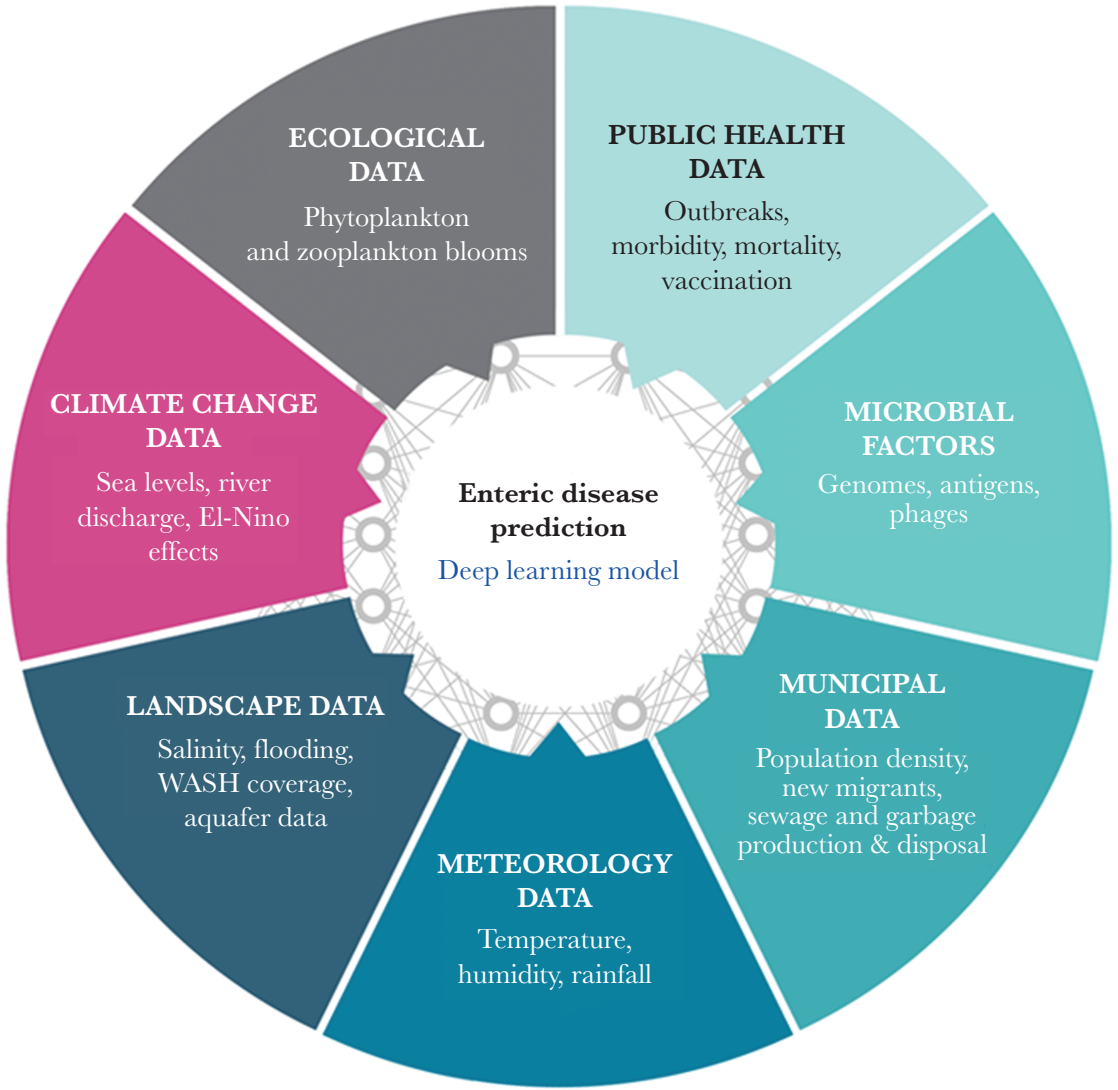
A suggested comprehensive approach towards developing a prediction model for enteric diseases through deep learning. Data from multiple domains, including environment and the public health sectors coupled with knowledge of microbial factors, can enable the development of an efficient multisectoral model that could help predict the onset of enteric diseases, such as cholera, whose endemicity and seasonality depend on sociodemographic variables and environmental flux, apart from host and microbial factors. Such a model could also be extended to the analyses of the onset and transmission of other enteric diseases such as salmonellosis as well as the emerging pandemic of antimicrobial resistance. Abbreviation: WASH, water, sanitation, and hygiene.

Utilizing whole-genome sequences along with phenotypic data can provide crucial information on the transmission as well as underlying mechanisms adopted by the superbugs at various levels—strains (prevalent lineages), genomic mutations/acquired genes (diversity of AMR mechanisms), and vehicles (diversity of MGEs harboring resistance genes) [27]. Whole-genome sequence-based systematic surveillance of pathogens can play a significant role in the detection and tracking of the emergence and transmission of high-risk bacterial clones, as well as significantly contribute to the development and modification of clinical practice guidelines [27]. Expansion of these efforts into middle- and low-income countries is also crucial to achieve effective surveillance and tackle the spread of AMR at a global level. Being an issue of multifaceted nature, a one-health approach based on the fact that human health is linked with animal health as well as the environment [28], is warranted for addressing the concerns of AMR. Finally, we suggest that interdisciplinary data science approaches, such as developing a deep learning model (Figure 2) leveraging multidomain data (from public health facilities, municipal settings, meteorology, climate change, irrigation and land use, as well as the microbial genomes, antigens, phage ecology, etc.), can be employed in order to comprehensively understand the onset of enteric diseases and AMR emergence patterns, and devising action plans towards mitigation. This is particularly relevant in the case of cholera and typhoid, the two diseases that follow a significant seasonality [2] and depend on environmental flux as well as host population dynamics. With significant modularity, such a model could be adopted for the prediction of other infectious diseases and as a crucial step towards mitigation of the foreseen pandemic of AMR. Finally, we advise that (1) a comprehensive and coordinated surveillance of AMR including the FMIE across the human, animal, and the environmental interfaces; (2) devising early warning systems as suggested in Figure 2; (3) establishing measures to curb the overuse and misuse of antimicrobial agents; and, (4) minimizing the use of antibiotics in food animals are crucial for tackling the global problem of AMR, which is growing as another pandemic in the offing while the entire gamut of attention, and resources currently remain focused on coronavirus disease 2019 (Covid-19).

## Notes

***Acknowledgment.*** We thank Ramani Baddam for providing images for Figure 1 and summarizing therein her analyses of the antimicrobial resistance encoding genes in the context of plasmid incompatibility groups.

***Financial support.*** This work was supported by the International Centre for Diarrheal Disease Research, Bangladesh (core grants) and the University of Hyderabad (support under IoE-PDF); Indian Council of Medical Research (postdoctoral research associateship to S. S. and grant vide AMR/257/2021/ECD-II to N. A.); Council of Scientific and Industrial Research, India (Junior Research Fellow/Senior Research Fellow to A. S.); and Microsoft Corporation (Microsoft Azure for Research and AI for Earth awards to N. A. and subsequent, on-demand Azure sponsorships for analyses).

***Potential conflicts of interest.*** All authors: No reported conflicts of interest. All authors have submitted the ICMJE Form for Disclosure of Potential Conflicts of Interest. Conflicts that the editors consider relevant to the content of the manuscript have been disclosed.

## References

[1] Ahmed N . A flood of microbial genomes-do we need more? PLoS One 2009; 4:e5831. 19513110 10.1371/journal.pone.0005831 PMC2688083

[2] Baddam R, Sarker N, Ahmed D, et al Genome dynamics of *Vibrio cholerae* isolates linked to seasonal outbreaks of cholera in Dhaka, Bangladesh. MBio 2020; 11:e03339-19. 32047137 10.1128/mBio.03339-19 PMC7018647

[3] Kumar N, Mariappan V, Baddam R, et al. Comparative genomic analysis of *Helicobacter pylori* from Malaysia identifies three distinct lineages suggestive of differential evolution. Nucleic Acids Res 2015; 43:324–35. 25452339 10.1093/nar/gku1271 PMC4288169

[4] Shaik S, Ranjan A, Tiwari SK, et al Comparative genomic analysis of globally dominant ST131 clone with other epidemiologically successful extraintestinal pathogenic *Escherichia coli* (ExPEC) lineages. MBio 2017; 8:e01596-17. 29066550 10.1128/mBio.01596-17 PMC5654935

[5] Ranjan A, Shaik S, Nandanwar N, et al Comparative genomics of *Escherichia coli* isolated from skin and soft tissue and other extraintestinal infections. MBio 2017; 8:e01070-17. 28811343 10.1128/mBio.01070-17 PMC5559633

[6] Hussain A, Ranjan A, Nandanwar N, Babbar A, Jadhav S, Ahmed N. Genotypic and phenotypic profiles of *Escherichia coli* isolates belonging to clinical sequence type 131 (ST131), clinical non-ST131, and fecal non-ST131 lineages from India. Antimicrob Agents Chemother 2014; 58:7240–9. 25246402 10.1128/AAC.03320-14 PMC4249578

[7] Ranjan A, Shaik S, Mondal A, et al. Molecular epidemiology and genome dynamics of New Delhi metallo-β-lactamase-producing extraintestinal pathogenic *Escherichia coli* strains from India. Antimicrob Agents Chemother 2016; 60:6795–805. 27600040 10.1128/AAC.01345-16 PMC5075088

[8] Ranjan A, Shaik S, Hussain A, et al. Genomic and functional portrait of a highly virulent, CTX-M-15-producing H30-Rx subclone of *Escherichia coli* sequence type 131. Antimicrob Agents Chemother 2015; 59:6087–95. 26195517 10.1128/AAC.01447-15 PMC4576125

[9] Merker M, Tueffers L, Vallier M, et al. Evolutionary approaches to combat antibiotic resistance: opportunities and challenges for precision medicine. Front Immunol 2020; 11:1938. 32983122 10.3389/fimmu.2020.01938 PMC7481325

[10] Baker S, The HC. Recent insights into *Shigella*: a major contributor to the global diarrhoeal disease burden. Curr Opin Infect Dis 2018; 31:449–54. 10.1097/QCO.0000000000000475 PMC6143181 30048255

[11] Ahmed N, Dobrindt U, Hacker J, Hasnain SE. Genomic fluidity and pathogenic bacteria: applications in diagnostics, epidemiology and intervention. Nat Rev Microbiol 2008; 6:387–94. 18392032 10.1038/nrmicro1889

[12] Sarker N, Shaik S, Baddam R, Ahmed N. Molecular epidemiology and genomics of multiple drug resistant bacteria in India and Bangladesh: a South Asian perspective. ASM Microbe, San Francisco, CA, 2019.

[13] Ahmed N . Beyond antibiotics. XXXIInd International Workshop on Helicobacter and Microbiota in Inflammation and Cancer, European Helicobacter Microbiota Study Group, Innsbruck, Austria, 2019.

[14] Vrancianu CO, Popa LI, Bleotu C, Chifiriuc MC. Targeting plasmids to limit acquisition and transmission of antimicrobial resistance. Front Microbiol 2020; 11:761. 32435238 10.3389/fmicb.2020.00761 PMC7219019

[15] Qaria MA, Qumar S, Sepe LP, Ahmed N. Cholesterol glucosylation-based survival strategy in *Helicobacter pylori*. Helicobacter 2021; 26:e12777. 33368895 10.1111/hel.12777

[16] Verma J, Bag S, Saha B, et al. Genomic plasticity associated with antimicrobial resistance in *Vibrio cholerae*. Proc Natl Acad Sci U S A 2019; 116:6226–31. 30867296 10.1073/pnas.1900141116 PMC6442563

[17] Das B, Verma J, Kumar P, Ghosh A, Ramamurthy T. Antibiotic resistance in *Vibrio cholerae*: understanding the ecology of resistance genes and mechanisms. Vaccine 2020; 38(Suppl 1):A83–92. 31272870 10.1016/j.vaccine.2019.06.031

[18] Suresh A, Shaik S, Baddam R, et al Evolutionary dynamics based on comparative genomics of pathogenic *Escherichia coli* lineages harboring polyketide synthase (Pks) island. MBio 2021; 12:e03634-20. 33653937 10.1128/mBio.03634-20 PMC8545132

[19] Rozwandowicz M, Brouwer MSM, Fischer J, et al. Plasmids carrying antimicrobial resistance genes in Enterobacteriaceae. J Antimicrob Chemother 2018; 73:1121–37. 29370371 10.1093/jac/dkx488

[20] Islam MA, Islam M, Hasan R, et al Environmental spread of New Delhi metallo-β- lactamase-1-producing multidrug-resistant bacteria in Dhaka, Bangladesh. Appl Environ Microbiol 2017; 83:e00793-17. 28526792 10.1128/AEM.00793-17 PMC5514672

[21] Mahmud ZH, Kabir MH, Ali S, et al. Extended-spectrum beta-lactamase-producing *Escherichia coli* in drinking water samples from a forcibly displaced, densely populated community setting in Bangladesh. Front Public Health 2020; 8:228. 32626677 10.3389/fpubh.2020.00228 PMC7314906

[22] Hussain A, Shaik S, Ranjan A, et al. Genomic and functional characterization of poultry *Escherichia coli* from India revealed diverse extended-spectrum β-lactamase-producing lineages with shared virulence profiles. Front Microbiol 2019; 10:2766. 31849903 10.3389/fmicb.2019.02766 PMC6901389

[23] Qumar S, Majid M, Kumar N, et al Genome dynamics and molecular infection epidemiology of multidrug-resistant *Helicobacter pullorum* isolates obtained from broiler and free-range chickens in India. Appl Environ Microbiol 2017; 83:e02305–16. 27815276 10.1128/AEM.02305-16 PMC5165125

[24] MacFadden DR, McGough SF, Fisman D, Santillana M, Brownstein JS. Antibiotic resistance increases with local temperature. Nat Clim Chang 2018; 8:510–4. 30369964 10.1038/s41558-018-0161-6 PMC6201249

[25] Ghosh C, Sarkar P, Issa R, Haldar J. Alternatives to conventional antibiotics in the era of antimicrobial resistance. Trends Microbiol 2019; 27:323–38. 30683453 10.1016/j.tim.2018.12.010

[26] Sockett RE, Lambert C. *Bdellovibrio* as therapeutic agents: a predatory renaissance? Nat Rev Microbiol 2004; 2:669–75. 15263901 10.1038/nrmicro959

[27] Argimón S, Masim MAL, Gayeta JM, et al. Integrating whole-genome sequencing within the national antimicrobial resistance surveillance program in the Philippines. Nat Commun 2020; 11:2719. 32483195 10.1038/s41467-020-16322-5 PMC7264328

[28] Robinson TP, Bu DP, Carrique-Mas J, et al. Antibiotic resistance is the quintessential one health issue. Trans R Soc Trop Med Hyg 2016; 110:377–80. 27475987 10.1093/trstmh/trw048 PMC4975175

